# Supplementary material for: Pathophysiological Investigation of Skeletal Deformities of Musculocontractural Ehlers–Danlos Syndrome Using Induced Pluripotent Stem Cells
Source: Genes (Basel). 2023 Mar 16;14(3):730. doi: 10.3390/genes14030730 (PMC10048181; doi:10.3390/genes14030730)
Supplement: Supplementary file 1 [file genes-14-00730-s001.zip › genes-2220359-supplementary.pdf]

Supplement Figure S1-S3. Osteogenic differentiation using normal iPSC line 253G1.

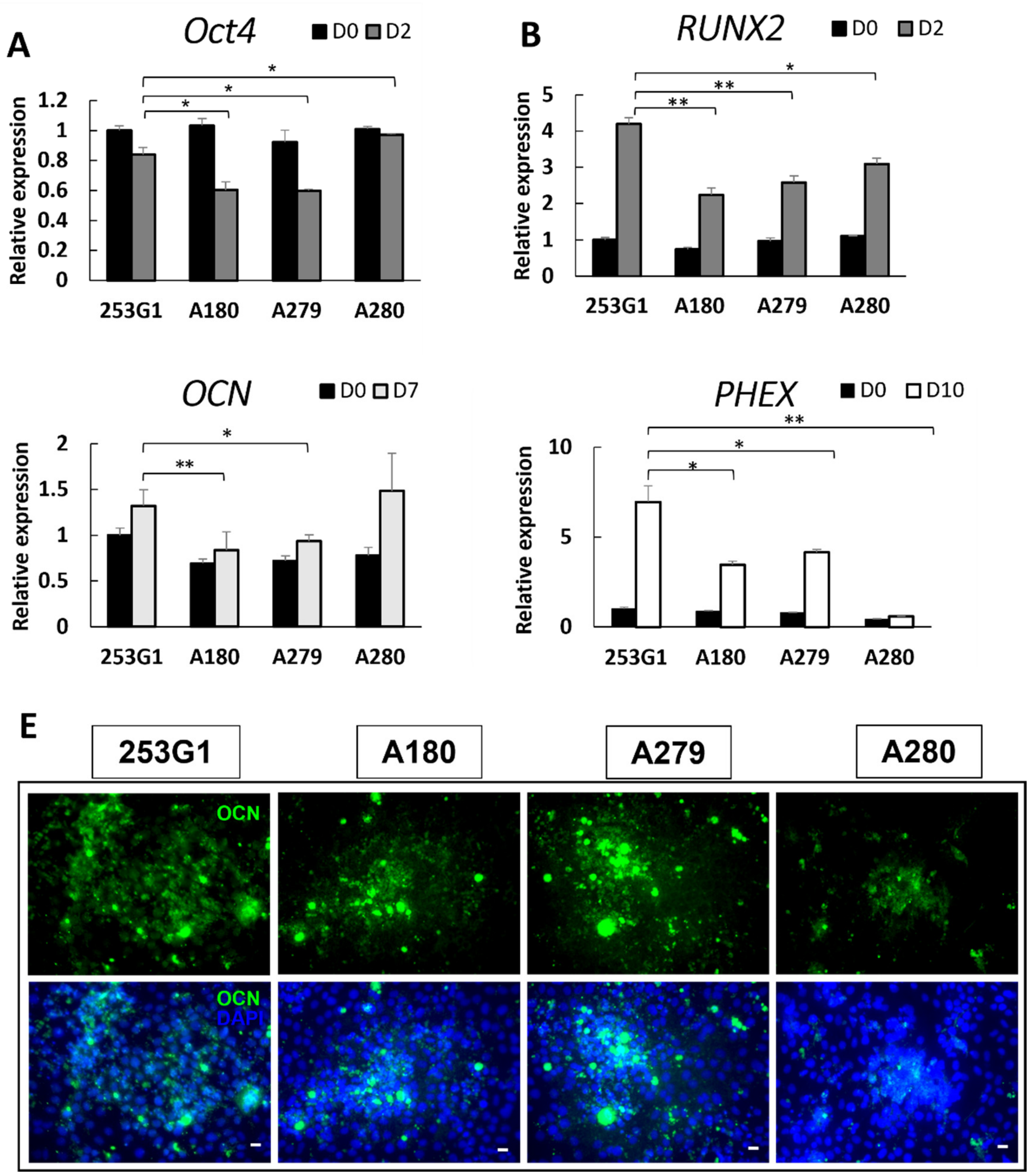

Figure S1. Differentiation of iPSCs into osteogenic lineages. (A–D) Quantification of the expression of undifferentiated pluripotent stem cell marker *OCT4* and osteogenic differentiation stage-related genes *RUNX2*, *OCN*, and *PHEX* at the indicated day, expressed as a value relative to that of WT iPSCs at Day 0. (E) Immunostaining of osteoblast-specific protein OCN (green) at Day 7. The presence of the cells was confirmed by staining of nuclei with DAPI (blue). Scale bars, 20 μm. Data expressed as the mean ± SD ($n = 3$); *$P < 0.05$, **$P < 0.01$.

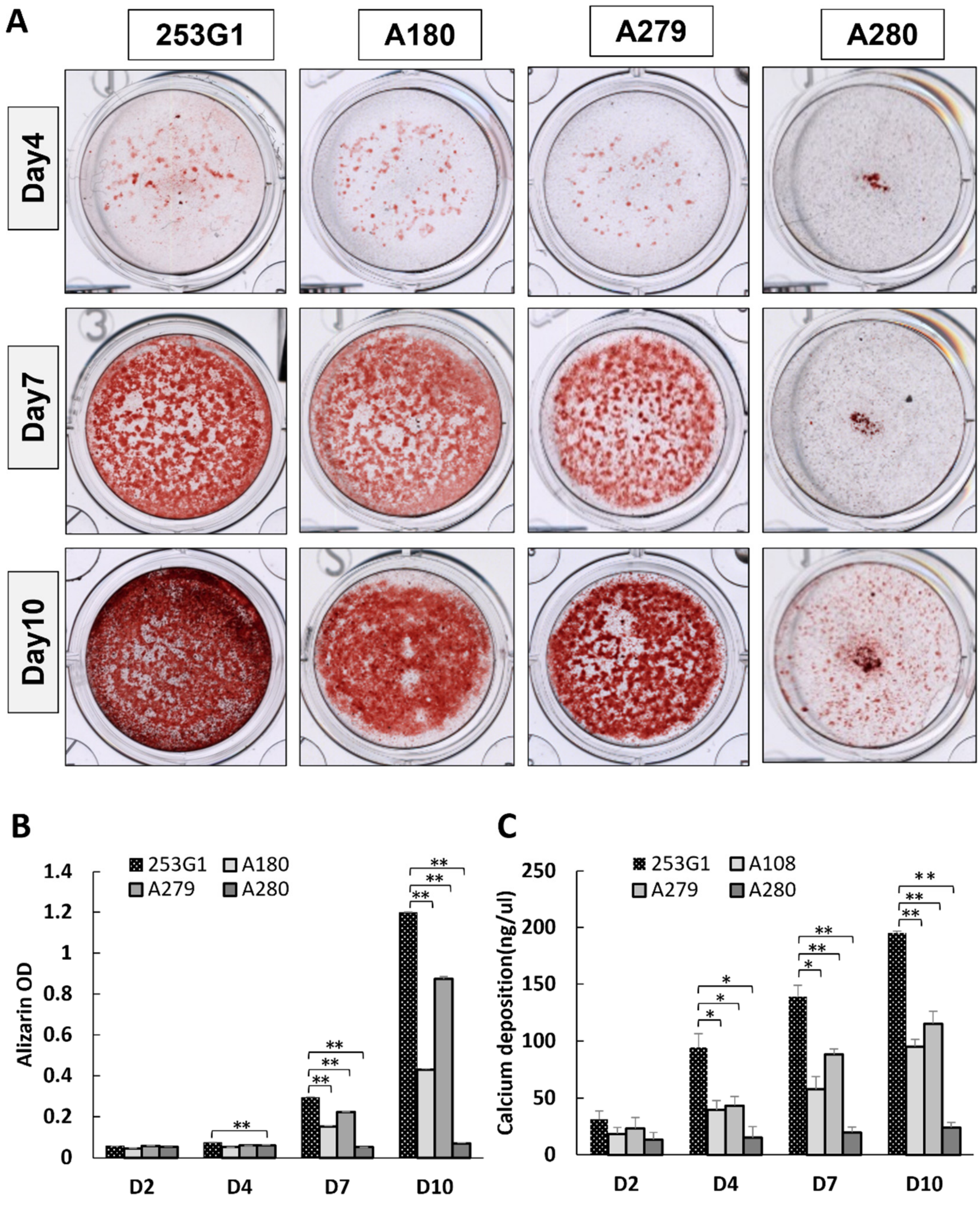

Figure S2. Impaired osteogenesis in mcEDS-CHST14 iPSCs. (A) Representative images of alizarin red staining (red granules) in WT and mcEDS-*CHST14* iPSCs during osteogenic differentiation. Evaluation of bone formation at the indicated day was quantified with dissolved alizarin solution (B) and the deposition of calcium (C). Results were normalized to cell number. Data expressed as the mean ± SD (n = 3); *P < 0.05, **P < 0.01.

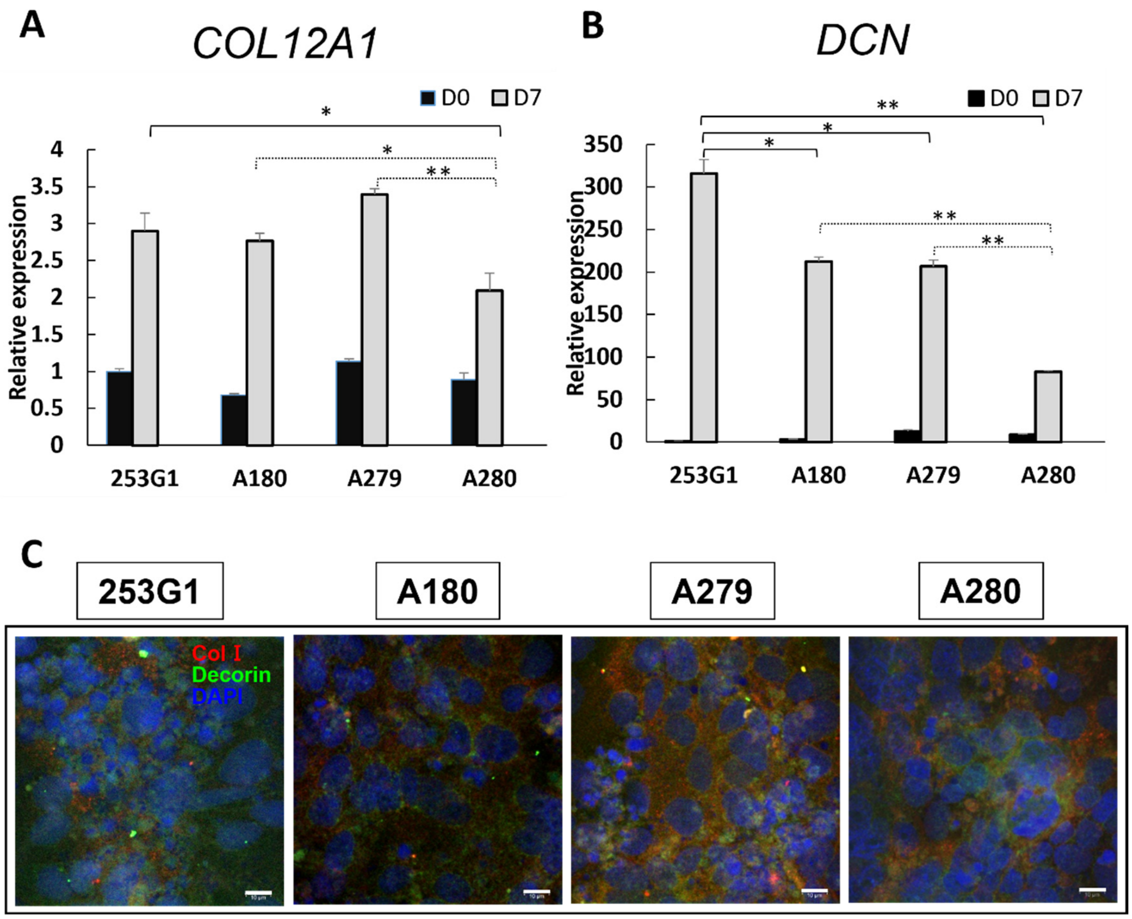

Figure S3. Expression of decorin and collagen in mcEDS-*CHST14* iPSC-derived osteogenic lineages. (A, B) Gene expression of collagen (*COL12A1*) and decorin (*DCN*) at Day 7, expressed as a value relative to that of WT iPSCs at Day 0. (C) Immunostaining of type I collagen (red) and decorin (green) at Day 7. Nuclei were counterstained with DAPI (blue). Scale bars, 10 µm. Data expressed as the mean ± SD (n = 3); *P < 0.05, **P < 0.01.

Supplement Table

Table.S1 Primers for sanger analysis

Primer	Sequence
M13-CH1	F: 5′-TGTAAAACGACGGCCAGTCCC ACCCCTTGAGCACCA-3′
	R: 5′-CAGGAAACAGCTATGACCCTTCATCACC CGCTTCCAGTTAGAG-3′
M13-CH2	F: 5′-TGTAAAACGACGGCCAGTGTTACCGCTT CCTCTACTGCTACG-3′
	R: 5′-CAGGAAACAGCTATGACCGGAAATCGG ACGTGAGGTGGTG-3′
M13-CH3	F: 5′-CAGGAAACAGCTATGACCGCACTATGAC TTTGTGGGCTCCTAT-3′
	R: 5′-TGTAAAACGACGGCCAGTCCAGAGCC AGAGTTTCTCCGAATG-3′
